# Lickometry: A novel and sensitive method for assessing functional deficits in rats after stroke

**DOI:** 10.1177/0271678X16684141

**Published:** 2017-01-06

**Authors:** Jewel Ahmed, Dominic M Dwyer, Tracy D Farr, David J Harrison, Stephen B Dunnett, Rebecca C Trueman

**Affiliations:** 1 School of Life Sciences, University of Nottingham, UK; 2 School of Psychology, Cardiff University, Wales, UK; 3 Brain Repair Group, School of Biosciences, Cardiff University, UK

**Keywords:** Animal models, behaviour (rodent), experimental, focal ischaemia, stroke

## Abstract

The need for sensitive, easy to administer assessments of long-term functional deficits is crucial in pre-clinical stroke research. In the present study, we introduce lickometry (lick microstructure analysis) as a precise method to assess sensorimotor deficits up to 40 days after middle cerebral artery occlusion in rats. Impairments in drinking efficiency compared to controls, and a compensatory increase in the number of drinking clusters were observed. This highlights the utility of this easy to administer task in assessing subtle, long-term deficits, which could be likened to oral deficits in patients.

## Introduction

There is a striking lack of novel stroke therapeutics, which has been partially attributed to experimental design of preclinical and clinical trials.^1,2^ One factor highlighted in preclinical research is the necessity to assess long-term functional outcome. Two to three weeks is recommended by The Stroke Treatment Academic Industry Roundtable (STAIR) criteria to demonstrate long-term benefits of putative therapies.^3^ From studies that have assessed long-term sensorimotor deficits,^4–8^ few tests are robust and sensitive past the first few weeks, particularly in animals with modest lesions. Tests highlighted as sensitive to chronic deficits focus on skilled motor abilities.^9–15^ While sensitive, many require extensive training or analysis, which is not always logistically feasible in a complex therapeutic study.

The lateral striatum, often damaged during middle cerebral artery occlusion (MCAO), is important for control of oral movements.^16^ Therefore, we present a straightforward test that requires little training and is sensitive to long-term deficits in oral function: lickometry. Licking is a stereotyped movement with little variability. Lick volume indicates the amount of fluid consumed per lick and is a measure of drinking efficiency.^17^ Drinking can then be broken down into clusters of licking, which require central pattern generators in the brain stem^18^ and are modulated by cerebellar inputs,^19^ as well pyramidal and non-pyramidal systems. Lickometry can assess the number of licks per cluster and the time between licks within a cluster (inter-lick interval (ILI)); increased variability in ILI is indicative of oral-motor dysfunction,^20^ as are changes in lick volume.^17^ Under standard (and non-pathological) conditions however, in order to consume more fluid, rodents do not alter lick volume, they increase the number of licks and reduce pauses between clusters of drinking.^21^

Lickometry is also highly translational as facial weakness and lingual deficits are present in patients. Nearly 45–55% of stroke patients who suffer from cortical or large subcortical stroke have alterations in tongue function, which is correlated with central facial palsy,^22^ dysarthria^23^ and the potentially life-threatening condition of dysphagia.^24,25^ This is not surprising considering the essential role of the tongue in speech and swallowing. Assessing rodent oral/tongue function in a high throughput, unbiased, quantitative manner, could provide a translational readout for treatment studies, which takes advantage of natural behaviours.

## Materials and methods

For full methods, please see the supplemental information. The present data are an in-depth analysis of lickometry, which was briefly presented as an outcome in Trueman et al.^10^ As that study demonstrated that ECA transection confounds behavioural measures and worsens welfare measures, for the current analysis, MCAO animals without ECA transection are compared to the combined naïve and sham groups (without ECA transection). For full details, please see Trueman et al.^10^ Experiments were performed according to the UK Animals (Scientific Procedures) Act, under Home Office authority and reported following the ARRIVE guidelines (please see supplemental methods for full details, including exclusion criteria). Thirty to forty days after surgery 22 male Wistar rats, who had undergone 60 min of MCAO via intraluminal filament method, 20 shams and 10 naïve rats were tested in automated drinking chambers with standard water bottles (Med Associates Inc., Hampton, UK), for 15 min daily over 4 days. During this period, the rats were water restricted with water available for 2 h per day. No animals lost more than 15% of their free feeding body weight, nor was there a difference in weight loss between the groups during this period of restriction. Several parameters were assessed: volume of water consumed per 1000 licks (lick volume), total water consumption, number of licks per drinking cluster, total number of clusters, total number of licks, ILI, and ILI variability. For detailed methods, see supplemental information and literature.^26,27^ Two weeks after lickometry, the total amount of rat chow consumed in 1 h was also assessed. Rats were perfused between 90 and 100 days, brain sections stained with NeuN (1:4000, Chemicon, UK). Intact striatal volume and brain atrophy was assessed using ImageJ.

These data were compared to other behavioural measures taken in the original study at 28 days after MCAO, for details of the statistical analysis, and these behavioural measures, see the supplementary information.

## Results

**Figure 1.**
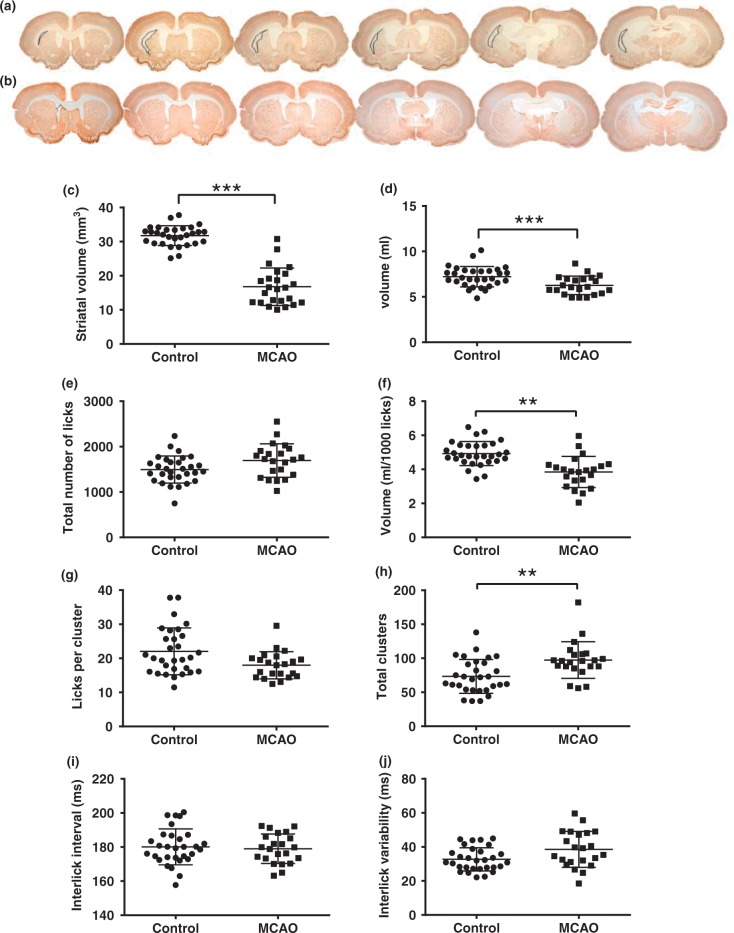
Representative NeuN stained sections from the median animal of the MCAO group, with the lesion outlined (a) and control group (b). (c) Ipsilateral striatal volume between 90 and 100 days post surgery. (d) Amount of fluid consumed, (e) total number of licks, (f) lick volume, (g) number of licks per cluster, (h) total number of clusters, (i) average ILI, (j) inter-lick variability (controls, n = 30 and MCAO, n = 22). The data are presented as mean and standard deviation. ** indicates p values of < 0.01 and ***p values of < 0.001.

**Figure 2.**
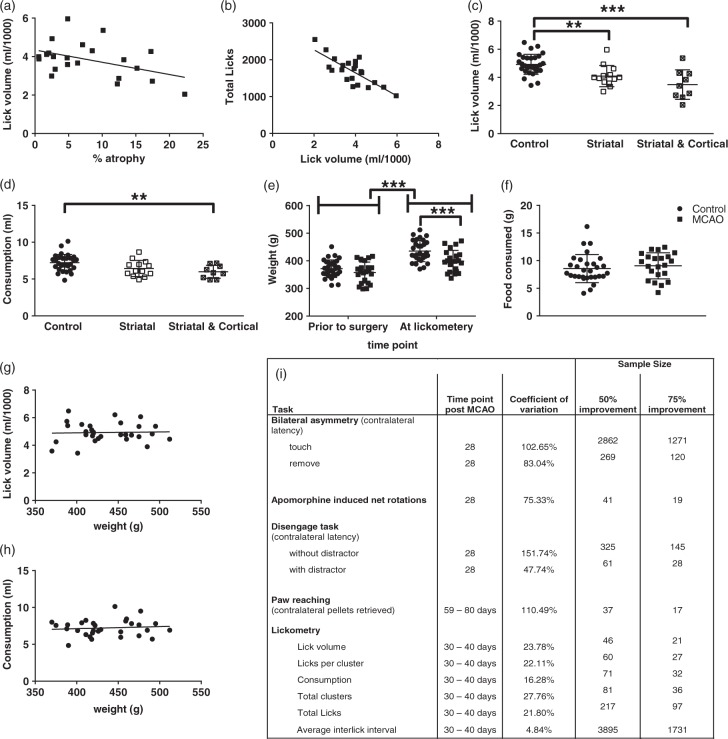
Lick volume correlated with brain atrophy (a) and total number of licks (b) for the MCAO animals (n = 22). Lick efficiency (c) and total water consumption (d) in control and MCAO animals with striatal only (n = 13) and striatal and cortex lesions (n = 9). (e) Body weight of all rats prior to surgery and at time of lickometry (30–40 days post-MCAO). (f) Food consumption in 1 h, 2 weeks following lickometry. Body weight did not correlate with lick efficiency (g) or total water consumption (h) in the control rats (n = 30). (i) The relative variability (measured by co-efficient of variation) of the lickometry measures in comparison to other behavioural tasks performed in the same group of rats (published in Trueman et al.^10^). Bilateral asymmetry (adhesive removal or sticky dot) measures the latency to contact and remove a sticky label from the forepaws. Apomorphine induced rotations counts the circling over 90 min following administration of 1 mg/kg of apomorphine. The disengage task measures the latency to respond to stimulation with and without a distractor (food). Paw reaching measures the number of pellets the rat is able to retrieve with each paw in staircase chambers. Sample size calculations were performed to establish the group sizes needed to see a treatment effect: 50% or 75% improvement (with control performance = 100%, MCAO group performance = 0%), based on the standard deviation of the MCAO group, alpha = 0.05 and power = 0.80. The data are presented as mean and standard deviation. ** indicates p values of < 0.01 and ***p values of < 0.001. Controls, n = 30 and MCAO, n = 22.

MCAO rats exhibited significant atrophy of the ipsilateral hemisphere (Figure 1(a)–(c)) and deficits in 3/7 lickometry parameters. MCAO resulted in a reduction in water consumption (t_50_ = 3.179, *p* = 0.0025, Figure 1(d)). This was not due to performing fewer licks; in fact, there was a trend towards MCAO rats performing more licks (Figure 1(e), t_50_ = 2.143, *p* = 0.04, ns due to Bonferroni). However, the volume consumed per 1000 licks was decreased in MCAO animals (Figure 1(f), t_50_ = 4.805, *p* = 0.0001), indicating a reduction in lick efficiency, which correlated with atrophy (Figure 2(a), r = −0.439, p < 0.05). The reduced lick efficiency also correlated with the increase in total licks (Figure 2(b), r = −0.649, p < 0.001), and likely reflects a compensation mechanism to maintain consumption due to reduced lick efficiency. When examining drinking patterns, there was a trend towards the MCAO group making fewer licks per cluster (U = 209, p = 0.02, ns due to Bonferroni) (Figure 1(g)), which was compensated by an increase in clusters (U = 169, p = 0.0024, Figure 1(h)). No differences were found in ILI (t_50_ = 0.404, p = ns, Figure 1(i)), or ILI variability (t_50_ = 2.422, p = ns, Figure 1(j)). When Bayes factors were calculated for all parametric measures, the only measure that supported the null hypothesis was ILI (Bayes factor, K = 4.44). There was strong support for the alternative hypothesis (a difference between MCAO and control) for consumption (K = 13.97), volume per lick (K = 1330.59) and ILI variability (K = 2.60) and weak support for total number of licks (K = 1.53).

For the key measures of lick efficiency and consumption, we divided MCAO rats into those with striatal only and striatal and cortical lesions. Both groups had significantly lower lick efficiencies (Figure 2(c), F_2,49_ = 13.61, p < 0.0001, striatal vs control: p = 0.0067, striatal and cortical vs control: p < 0.0001). However, only the striatal and cortical group reduced the volume of water consumed (Figure 2(d), F_2,49_ = 5.52, p = 0.0069, control vs cortical and striatal: p < 0.0116).

Although the MCAO rats had oral deficits, they maintained food intake. The MCAO rats consumed an equal amount food compared to the control group (Figure 2(f)) and the weight of all rats increased significantly from the day of surgery (Figure 2(e), F_1,50_ = 1004, p < 0.0001). However, at the time of lickometry, the MCAO animals were lighter than the controls (Figure 2(e), F_1,50_ = 45.25, p < 0.0001, MCAO vs control: p = 0.001). To ensure that differences in body weight did not influence consumption or lick efficiency, these measures were correlated with body weight in the control group and no relationship was found (Figure 2(g) and (h)).

The coefficient of variation for the lickometry measures was lower than other behavioural measure performed in the same group of rats (Figure 2(i)), and this resulted in acceptable sample sizes for potential treatment studies (Figure 2(i)).

## Discussion

The purpose of the present data was to demonstrate the usefulness of lickometry for assessing long-term post-stroke functional deficits. This test has translational potential, as lack of lingual coordination and deficits in tongue function are common presentations in stroke patients and are associated with dysphagia and dysarthria.^24,25^ The results are the first to suggest that MCAO leads to lasting deficits in drinking behaviour.

Peripheral sensory and motor denervation has been reported to produce a reduction in volume per lick, and an increased number of licks without changes in ILI.^17^ The present results are in agreement, demonstrating that this is most likely a sensorimotor deficit. While lick efficiency decreased in the MCAO group, the total number of licks was slightly increased, and there were more licking clusters without changes to ILI. The increased number of licks and clusters is likely an attempt to compensate for the decreased licking efficiency, and indicates MCAO rats do not have reduced motivation to drink despite weighing slightly less than the controls. Although body weight may influence overall daily consumption, it did not confound this task as it did not influence the amount of water consumed in this short 15 min test and it did not correlate with lick efficiency. Additionally, despite having very little variance (4.87% CV), the fact that the ILI was not affected, as supported by the Bayes factor, also indicates that this is a sensorimotor deficit, rather than an alteration in control of the drinking pattern. It should be highlighted that the deficits detected in these animals needed subtle analysis with equipment that could record to the 0.01 s, they did not have such significant impairments in oral function that they became malnourished or dehydrated.

Brain atrophy was correlated with lick efficiency, and a sub-analysis of striatal only versus striatal and cortical lesions indicated the decrease in consumption was only evident in the animals with striatal and cortical damage. This highlights the ability of this test to discriminate lesion types. However, lick efficiency was reduced in the animals with pure striatal lesions as well, demonstrating the sensitivity of this task to detect deficits in animals with modest lesions. One limitation of the present study is that the histology was not performed at the same timepoint as the lickometry, though we do not expect further lesion evolution beyond the 40-day period.

Previously, only tongue protrusion has been examined after MCAO.^28^ Lickometry provides a more comprehensive analysis of tongue function and is highly sensitive to long-term deficits. There is very low variation in the data obtained from this task in comparison to the other behavioural measures performed on the same rats. Furthermore, lickometry results in reasonable sample size predictions for treatment studies (n = 21), similar to that required for paw reaching, which is often reported as a sensitive test, and much less than the bilateral asymmetry task (or stick dot, n = 120), which is a commonly used test. Unlike many tests of sensorimotor function,^15^ drinking is a behaviour that the rats engage in multiple times a day and thus if spontaneous recovery and/or compensation was to occur, it would happen quickly following MCAO without needing exposure to the test itself. However, as deficits were still seen at 30–40 days, it is unlikely that compensation or significant natural recovery will be seen with this task.

In addition to the high sensitivity, as it is able to detect oral deficits in animals with modest lesions, lickometry has practical advantages over other behavioural tests. It can be fully automated, and it is quantitative. Drinking is a natural behaviour for animals, so very little training is required. Water restriction is required but only needs to be implemented for a few days (usually 4–5 days) and should not impact the health or welfare of the animals. Nevertheless, there are a few considerations with regards to implementation of a lickometry system. For example, the distance between the animal and the drinking tube can influence the licking rate, specifically, the amount of ‘tongue travel’ is negatively related to licking frequency.^29^ When using lickometry for stroke research, this should be carefully considered, as the animals may have postural difficulties. However, as we saw no alteration in ILI, this did not appear to be a problem with the set-up used in this study. The equipment presented here may appear costly, and labs must consider this when choosing to invest in automated systems. However, the reduction in bias and man-power might outweigh the initial cost of the equipment, and less expensive, simpler versions are available than the one presented here.

Overall, lickometry provides a reliable, quantitative measure of long-term oral deficits that is easy to implement in the rodent. Furthermore, this test is highly translational, as oral deficits, while largely unexplored in the rodent, are comparable to symptoms exhibited by the patient population, such as tongue protrusion and the corresponding dysarthria and dysphagia.

## Supplementary Material

Supplementary material
