# Supplementary material for: Lickometry: A novel and sensitive method for assessing functional deficits in rats after stroke
Source: J Cereb Blood Flow Metab. 2017 Jan 6;37(3):755–61. doi: 10.1177/0271678X16684141 (PMC5305038; doi:10.1177/0271678X16684141)
Supplement: Supplementary material [file JCB684141_supplementary_material.pdf]

Materials and methods

Animals and experimental design

Sixty-four male Wistar rats (10-12 weeks old; Harlan, UK) were housed in standard cages, in groups of four, within a temperature (21±2°C), humidity (55±10%), and light (14/10h light/dark cycle) controlled environment. Animals were randomised to groups and the experiments conducted in a blind fashion. There was a single mortality in the MCAO group, three rats were excluded due to subarachnoid haemorrhage during surgery (indicated by no restoration of blood flow on Laser Doppler, following removal of the filament) and 6 due to lack of visible lesion with MRI 24 hours after surgery. Animals that did not drink in the testing equipment were excluded from analysis (n=2, MCAO). This resulted in 22 rats in the MCAO and 30 rats (10 Naive and 20 sham) in the control group. This outcome measure had not been assessed in this model before, so sample size was based on other behavioural measures assessed in the original Trueman et al. 2011[1] study.

Surgery

Animals were anaesthetised using isoflurane in a 60:40 nitrous oxide: oxygen mixture, and core temperature was maintained at 36.7 ± 1.0°C using automated heat blankets (Harvard Apparatus, Kent, UK). A Laser Doppler probe was placed through the temporal muscle against the skull. Following exposure of the right carotid artery (CA), a filament (390 – 410μm, Doccol Corporation, Redlands, USA) was advanced, and withdrawn after 60min. Shams underwent the same procedure without filament introduction, naïve animals did not undergo anaesthetic. Rats were given 1mg/mL of paracetamol (Boots, Nottingham, UK) in the drinking water 1 day prior to and for 3 days after surgery. Animals received daily fluid replacement and moistened food until weight stabilized.

Lickometry

Rats were water restricted (water available for 2hrs in the afternoon) for 4 days between 30 and 40 days following surgery, licking behaviour was tested in automated drinking chambers (Med Associates Inc., Hampton, UK) for 15min daily (am). Water was available through stainless-steel drinking spouts attached to standard water bottles. A contact-sensitive lickometer measured each lick to the nearest 0.01s and a computer recorded the data. Water bottles were weighed before and after testing.

Several parameters were assessed: volume of water consumed per 1000 licks (lick volume), total water consumption, number of licks per drinking cluster, total number of clusters, total number of licks, ILI, and ILI variability. A maximum inter-cluster interval of 0.5 s was applied, if rats paused for longer than this between licks; a new cluster commenced. For detailed methods see[2,3].

Food consumption

Fourteen days after Lickometry, rats were food restricted by giving set amounts of food daily to reduce body weight to 80-90% of free feeding weight. On day 16 they were individually housed for 1h and the amount of normal rat chow consumed was assessed.

Other behavioral measures

During the original study the bilateral asymmetry (sticky label) and disengage tests were performed on days 1, 7, 28, and 90 post MCAO. Apomorphine induced rotation was conducted at 7, 28, and 90 post MCAO, and paw reaching was performed between days 59 and 80. The variability (via coefficient of variation) of the lickometry measures was compared to these other tasks at the 28 day time point (or 59 – 80 for the pawreaching).

Rotation. A subcutaneous injection of apomorphine (1mg/kg) was administered and the net number of ipsilateral rotations were recorded (number of turns clockwise – number

counterclockwise) for 90 mins by automated rotometers (Med Associates Inc, St Albans VT, USA).

Bilateral asymmetry. Squares of adhesive tape (7 x 7mm) were stuck on the anterior wrists of both forelimbs. The rats were placed in a Perspex cylinder (35cm high 32cm diameter) and the time (s) of first contact and removal for each forelimb was recorded.

Disengage. Using a cotton bud, the perioral region was stimulated on both side and latency to respond (s) was recorded. This test was repeated while the animals were eating chocolate, with the order of testing (with and without chocolate, left and right side) randomized.

Paw reaching. 55 days following MCAO surgery rats were food restricted so that body weight stabilized to 85-90% of the free feeding weight. On day 59 paw reaching training started and was carried out for 15 mins a day for 20 days. Rats were placed in a staircase chamber and each step was filled with five 45mg sucrose food pellets (Sandown Scientific, Hampton, UK). Total number of food pellets taken with each forelimb was recorded.

Histology

Between 90 and 100 days, rats were anaesthetized with Euthatal (Merial Animal Health Ltd., Harlow, UK), and perfused with phosphate buffered saline followed by 1.5% paraformaldehyde. Brains were post-fixed for 24h, cryo-protected in 25% sucrose and sectioned to 40μm on a freezing sledge microtome (Leica, Wetzlar, Germany), and a 1/12 series was stained for NeuN (1:4000, Chemicon, UK) using diaminobenzidine (DAB). Sections from the most anterior tip to the most posterior end of the striatum were photographed. Ipsilateral intact striatal volume was measured, and atrophy was assessed using ImageJ and the equation [(area of ipsilateral hemisphere - area of contralateral hemisphere)/ area of contralateral hemisphere]*100.

Statistical analysis

Data from the final day of lickometry was analysed using Prism. No differences were found between sham and naïve animals, so they were combined into 1 control group. Data was tested for normality (D'Agostino & Pearson omnibus) and analysed using unpaired t-tests and one or two-way ANOVAs, or a Mann-Whitney test. Bonferroni corrections were applied to comparisons between MCAO and control for the lickometry data resulting in $\alpha = p < 0.007$. In addition, for parametric data Bayes factors were calculated to assess support for both the null and alternative hypothesis using version 0.9.8 of the BayesFactor package[4]. Pearson's correlation was carried out between histological, behavioural, and weight measures. Sample size estimations were calculated using PS sample size and power calculation software version 19 with power = 0.8 and $\alpha = 0.05$.

References

1. Trueman RC, Harrison DJ, Dwyer DM, et al. A Critical Re-Examination of the Intraluminal Filament MCAO Model: Impact of External Carotid Artery Transection. *Transl Stroke Res* 2011; 2: 651–661.

2. Dwyer DM, Lydall ES, Hayward AJ. Simultaneous contrast: evidence from licking microstructure and cross-solution comparisons. *J Exp Psychol Anim Behav Process* 2011; 37: 200–10.

3. Wright RL, Gilmour G, Dwyer DM. Microstructural analysis of negative anticipatory contrast: A reconsideration of the devaluation account. *Learn Behav* 2013; 41: 353–9.

4. Rouder JN, Speckman PL, Sun D, et al. Bayesian t tests for accepting and rejecting the null hypothesis. *Psychon Bull Rev* 2009; 16: 225–37.
